# Editorial

**Published:** 1859-03

**Authors:** 


### Editorial.

### THE AMERICAN JOURNAL.

The *American Journal* has two grave objections to controversy; first, because there is danger of becoming involved in "endless and unprofitable controvery;" second, because the majority of readers have the opportunity to read but one side of the question, which is not always satisfactory. The *grave* reasons are good. He has also one which he considers "graver," the import of which seems to be that he has more water in him than any of those whom he is likely to meet in combat. We do not know how else he applies his quotation from the "Autocrat," which is as follows:

"If a fellow attacked my opinions in print, would I reply? Not I. Do you think I don't understand what my friend, the Professor, long ago called *the hydrostatic paradox of controversy?* Don't know what that means? Well, I will tell you. You know, that, if you had a bent tube, one arm of which was the size of a pipe-stem, and the other big enough to hold the ocean, water would stand at the same height in the one as in the other. Controversy equalizes fools and wise men in the same way—*and the fools know it.*"

The "Journal" objects to controversy, because it does not choose *to put himself on a level* with every individual of a certain class. He *knew* his position was correct—that the tendency is to *level*, but, lest others might doubt its validity, he quotes the "Autocrat" to prove it, and as his authority, in speaking of this leveling tendency, says, "*the fools know it.*" We infer that we have found the "Autocrat" fool, self-confessed, in the editor of the "Journal." But, as we are "candid" now, we presume the confession is another "slip of a too hurried pen."

But it is perhaps, "natural for those who sojourn so near to the *broad Atlantic*, to boast of their "water privileges" and to regard all others as the necessary patrons of "horse power" and hand-mills. But we advise all such to be reasonable in their assumptions, lest the stump-orator's threat be put in execution. "Who keers," says he, "for the mistress of the ocean? Aren't we the masters of it? There wouldn't have been any ocean if it hadn't been for the old Mississippi. And if we dig a ragin' canawl and turn that big drink into the mammoth cave, then wars your ocean? *It'll bile dry, sir.*"

The editor objects to what he terms our "scurrilous personalities" in a former article. We beg his and the reader's pardon for using them; and to show that we have reformed, under the influence of the courteous and chaste example he has set us in his present article, we will not call him a "scribbler," nor charge him with dishonorable notoriety, nor speak of his "chemical quackery" (unless we want to), nor allude to his fund of ignorance," nor say that he is not a chemist, nor write a great many phrases which would be "scurrilous" if we used them, but which are chaste and gentlemanly when used by the editor of the Journal.

But the editor says we charged him with *jealousy*. His memory is bad. We defended him against the charge, and claimed that he was actuated by the purest motives, and impelled by stern necessity, as will appear by reference to our article, Vol. XI, p. 465, of the "Register."

The editor's worst objections to us, as we infer from his emphatic repetition of it, is that we are not acquainted with Bischoff. He might pardon our other sins, on due repentance and reformation, but that one he can not forgive. The charge, we admit, is a serious one, but we are gratified to think that the two unsettled questions now pertain to chemistry. "Who struck Billy Patterson" is ruled out, for the "rappers," by disquieting his spirit, have learned that he never was struck; and the questions are now, "will saltpetre explode?" and do you know Bischoff?"

"In the last paragraph but one of" the editor's "article we have the extorted confession that" cane sugar is not the only variety used in food; and we would not have noticed this "slip of a too hurried pen," if the editor had not ignored the fact that by the first step in fermentation, cane sugar is changed to fruit sugar.

The editor brings "these solemn exercises" to a close with this sad and sensible paragraph:

"It is not likely that we will soon again step out of our way to notice W. He attracted our attention in the first instance by meddling with things above his comprehension. We should not then have noticed him publicly, were it not that his chemical crudities were paraded as a part of the proceedings of one of our societies, that we suspected they would be reproduced in Europe, and that we anticipated a storm of ridicule which would fall not upon him, but upon the whole dental profession in America. To avert this by showing the world they were not received here, was the sole object of our first article. *Ne sutor ultra crepidam* is a sensible old adage, which would save much trouble if generally acted on, and which we commend to W's serious consideration."

Not likely soon to notice us again! Now, doctor, that's cruel. We ought to be intimate; for there is a bit of romance connected with our earliest acquaintance. The editor says of us, "He attracted our attention, in the first instance, by meddling with things above his comprehension." Now, that attraction was reciprocated, and from like causes. ' 'We had no consciousness of his (the editor's) existence, till his chemical quackery" induced him to *attempt* to write a *dental* chemistry, without any practical knowledge of the wants of the dental profession; and we should not thus notice the fact " were it not that his chemical crudities" are "paraded as a part of" the literature of the profession. Our early unconsciousness of the editors existence is *meaner*, we know, than our present non-acquaintance with Bischoff, but as we were young then we hope to be forgiven.

The editor's apology for getting into the big arm of the "Autocrat's" bent tube, while we were in the little one, is frank and satisfactory. He was afraid our ideas would get across to Europe, and the whole profession in America would be overwhelmed with a storm of ridicule. Gracious man! he averted "this by showing the world that they were not received here." By the term world we suppose he means the few hundreds who read the "Journal"; and he showed these few *he* didn't receive them. That is all it amounts to,—but, simple as it is, it averted a storm more fierce and fearful than that one we read of

> "When the blue waves rolled nightly on the deep Galilee."

Verily, Professor Espy is no longer THE storm King.

Doctor, couldn't you establish branch offices at the other Atlantic ports? Some "crudities" may ship for Europe at other points, knowing that you are on the watch, at Baltimore to arrest them as fillibusters.

"*Ne sutor ultra crepidam*" is a sensible old adage——which we commend to the editor's "serious consideration." If this teaches that a *cobbler* should not get beyond his *last*, let us enquire which one of us has *stuck to his own last* the most faithfully. The Junior editor of the "Journal" is Professor of *anatomy and physiology*, in the Dental College at Baltimore, and formerly held the *same* chair in a Medical College. He left *his own last* and tried cobbling on that of a special chemist. It is not strange that the job, when finished, failed to fit the foot of the profession it was made for. On the other hand, we were selected, by the unanimous vote of one of the largest dental associations in the world, to teach DENTAL *Chemistry*, in the first building ever erected expressly for dental education, and we have never spoken or written any thing on *dental* chemistry, but at the request of some of our constituents or pupils.

We have stuck to our last, and are ready to resign it as soon as our patron can find a *cobbler* that will better answer her purpose.

Near the close of his article the editor exhibits a decided streak of frail humanity. When relieved of any ill which flesh inherits, the patient is loud in his praises of the remedy and its inventor. Hence we have numerous certificates of marvelous cures. As a race, we are all in search of something "to cure our ignorance;" and the editor, having obtained at least partial relief, is naturally anxious that all his friends should come to the same physician and be healed, hence he earnestly intreats us to call on an "apothecary's boy." We'll do it; for though the cure is but partial in his case, yet, considering how low the patient had sunk, we consider the success a triumph of science and skill. We would like to call on the same boy, if the editor would give us his address.

As we can find nothing new in the editor's objections to our views of the chemistry of caries, our readers will excuse us for neglecting to re-answer old ones. In the mean time, we will go on with our chemical investigations; and when we have any thing deemed worthy of notice, our readers, as usual, will hear from us. We would simply state that in all these remarks, concerning the editor of the "Journal," it is the JUNIOR who is referred to. We have too much respect for Professor Harris' age and talents to write in this style of levity concerning him; and he has too much respect for himself to do or say any thing that would call for such notice.

---

### VULCANITE BASE.

This style of work, though it has been used some two or three years East, has not, till very recently, attracted much attention in this city. Quite a number are now experimenting with it, with a view of ascertaining to what extent it is available as a base for artificial teeth. What will be the result, time only can determine. For the most part, where it has been longest used, the report is very favorable. Occasionally failures have been reported, but upon what conditions or circumstances they were predicated, is not definitely set forth. We have seen a number of pieces that have been worn in the mouth for two years, without undergoing any apparent change whatever. Indeed, we have yet to see a piece that has suffered any change in the mouth. Its durability is the great point to be tested; there is some objection to its color, but that is of minor importance, and scarcely enters into the account at all, when gum teeth or blocks are used. Its color is no more objectionable than that of gold. There are several things that recommend it very much; the perfect accuracy of adaptation; if the model is accurate, the fit of the plate will be perfect, without a possibility of a mistake. The base is formed, and vulcanized, on the model, and there is no expansion or contraction. It is very light, the weight being much less than any other style of work, and that in some cases it is an important consideration, and it is not objectionable in any case. With it all the strength necessary for any case can be obtained. There is no metallic work in which the teeth are more firmly retained; if they are properly set, there is no possibility of them being displaced by ordinary usage. In falling, and striking any hard substance, there is less liability of breaking the teeth, than with the metallic base. There are no openings or interstices in it for the lodgment of foreign substances; it is as perfect in this respect as continuous gum work; than which we could scarcely desire any thing better.

The general expression of those who have tested both, is that it is much more pleasant and congenial in the mouth than any metallic plate.

It can be worn in the mouth with any metal that it is proper to put into the mouth, either in the form of fillings or plate.

It is not affected by mercurials, as most metallic plates are, especially gold and silver. Our experience is rather limited, not extending over six weeks.

The first case was a full set, for a gentleman who had worn a silver set for two months, being very much annoyed thereby; in consequence of oxydation of the plates, they would become perfectly black in twenty-four hours, and were almost intolerable, giving a metallic taste constantly.

A set on vulcanite base has been used now for several weeks with great comfort. There is not the least taste or odor, though they have been worn for four days, without being taken out of the mouth. There is not that metallic sound, by the occlusion of the jaws, experienced with other work.

Another full set, temporary, put in four days after the teeth were removed, are worn with comfort, much more than the patient expected. There is no taste or odor.

Another, a full upper set, inserted instead of a gold set, that had been worn in connection with a partial under set, on cheoplastic base, in consequence of which there had been a constant offensive odor and taste in the mouth.

The vulcanite set is worn instead of the gold, and without any taste or odor, and the food is masticated quite as well as with the gold set.

This style of work should be thoroughly tried by all the progressive part of the profession. It will, doubtless, answer well in all cases of temporary work, and, we think, promises more than this.

Please test it and send us your experience, especially in regard to indestructibility. T.

---

### SOMETHING NEW.

We have, within the last day or two, witnessed something novel in the way of mounting sets of teeth. It consists in a method of mounting and attaching the teeth to the plate, after it is swaged and fitted in the usual manner, without soldering, and without the ordinary backings, yet with a beautiful continuous lining, that fits as perfectly to the teeth as though it was cast, is perfectly smooth, and better finished than any ordinary backings can be. Can be made to take any desired form. The attachment is a metallic union, and yet is without heat that will affect the plate, consequently, there can be no springing. It can be done with gold or silver. All interstices are perfectly closed. It is accomplished in much less time than the ordinary work, is more beautiful, and seems to be firmer. The attachment is with pure gold, or gold of any desired carat, or silv. We have no experience with this kind of work, but intend to experiment with it as soon as opportunity affords. We speak of the work as we have seen it, and as it was described to us.

We can not see why the work is not all that is claimed for it. Time will develope more in regard to it. T.

---

### PAINLESS SURGERY.

Dr. Livingston, the celebrated African traveler, states that when torn by a lion he felt no pain. Was it because the wounds inflicted were trivial? Certainly not; for the shoulder was crushed, and the humerus fractured so severely that a false joint is the result. Then think of the contused and punctured wounds inflicted by the jaws and teeth of the animal. He was not in a state of stupor bordering on coma; for he was able to observe minutely the actions of the lion, and to notice all the surrounding circumstances. He was not, probably, laboring under a state of depressed nervous action, for his energies were aroused, and he was the aggressor in the fight. Had he been fully under the influence of ether or chloroform, we would not be surprised to hear that he was unconscious of pain during the onslaught of the lion; but all the circumstances warrant the belief that his nervous system was in a very different, if not in the very opposite condition to that produced by chloroform.

But after all, is there any thing very uncommon in Dr. L.'s exemption from pain under such circumstances? In battles, mobs, or street fights, do not many receive severe wounds of which they are not conscious at the time? or if conscious, do they not fight on, without a sense of pain, even when disabled by fracture or loss of blood? Does not every rollicking school-boy know that when "fighting mad," a sound drubbing hurts him far less than when under a calmer mental condition?

Now, if we could devise a convenient method of rousing the nervous system up to the painless point, we would like it much better than the plan of depressing it, by anaesthetics, below the point of feeling. And if we could act successfully on only those nerves concerned, in any given operation, the result would be as satisfactory as if the whole nervous system were thus influenced. And something like this, we believe is accomplished by the use of a galvanic current in the extraction of teeth. It is needless to deny that, in many cases, the pain of the operation is very much lessened, if not totally abated; and we know of no better method of accounting for the fact than by the hypothesis above suggested. In short, we believe that the dental nerves, during a successful application of the battery, are in the same condition that Dr. Livingston's were when the lion had him. W.

---

"THE LEGS OF THE LAME ARE NOT EQUAL,"

Therefore, they are to be excused if they limp. The *American Journal*, on the same principle, is not to be held responsible for its flimsy explanation of its conduct in regard to the report of the proceedings of the Convention at Cincinnati. The lame man walks as well as he can, and the *Journal*, probably, gave the best excuse it had.

The editors call their report "a summary, furnished by a member, made up partly from recollection, and partly from advance sheets of the *Register*, and, we believe, of the *Reporter*, which he obtained."

As neither of the editors was at the meeting, they are excusable for *believing* any thing. But those present *know* that the gentleman who furnished their report, "obtained" no *Reporter*, and made no effort toward a systematic report himself. Everybody knows that his "recollection" would amount to but little without systematic notes; and as his report is only a *garbled*, *mutilated* copy of that which belonged, in common, to the *News Letter* and the *Register*, the public can judge of the *Journal's* candor.

As the editors "saw no other report till all the forms but one of the *Journal* were printed," it is probable that they were hoaxed, and supposed they had an *original report* instead of a purloined and mutilated *copy*. But why, then, defend their own course and that of their reporter? W.

---

A GAS EXPLOSION.

It is a commonly received opinion, that flame will not penetrate wire gauze or a tube having a small diameter. There is evidently some foundation for this belief, as the practical use of the "safety lamp" in mines will testify. But it is probable that far too much reliance may be placed in these appliances. Hemming's safety-tube is sometimes used to prevent the flame from reaching the reservoir of mixed gases, when experimenting with oxygen and hydrogen. This tube is made by filling a plain brass tube with fine wire, and wedging it tightly together, by forcing in a larger wire of a conical shape. A blowpipe point may then be attached to one end of it.

But we set out to tell the result of an experiment.

About a quart of oxygen and hydrogen, mixed in the proportions to form water, was contained in an elastic gas-bag. The bag was armed with a stop-cock, to which was connected a brass pipe five inches long, and a half inch in diameter. To this, an India-rubber tube, thirty inches long, was attached; and a safety tube, eight inches in length, with a very small orifice, terminated the apparatus.

The stop-cock was turned, and, by the pressure of the hand, the gas was forced out, and a match applied to the jet. While the gas was burning freely, the hand was gently raised, so as to reverse the current, and instantly a deafening explosion took place. We have no comments to make. W.

---

*Of Nature and Art in the Cure of Disease.* By Sir John Forbes. M. D., etc.

Who that has read Dickens' story of Bleak House, does not remember Turveydrop, the high-shouldered Model of Deportment? Well, Turveydrop has turned author, and written a book entitled as above. And a downright good book it is, too. The matter of it is excellent. Turveydrop undertakes to assert, in his high way, that the world is doctored to death. We confess, if we comprehend his genteel rhetoric, that we think so too. All the systems of medicine he says, have failed of their object. We subscribe—unless, indeed, their object has been to obviate a density of population. Nature, we understand him to intend to say, is the best physician in the world. We homologate. He recommends, if we are not mistaken, less medication and more reliance on the curative powers of the aforesaid physician. We indorse. In fine, and in earnest, Sir J. Forbes' book appears to be a reluctant confession, in very wordy, prolix, and pedantic diction, of an old doctor's disgust with doctoring and doctor-stuff. The book contains several wholesome truths—and several thousand words. Indeed, there is too much language in the volume, by half. No doubt, Sir J. Forbes has a confused notion of some aim he is driving at, but Sir J. Forbes never makes any center-shots at all. He may be talking at a mark, but he never talks *to* it. There is a wandering, wool-gathering, circumlocutory booziness in his style, as if Sir J. Forbes had had "a brick in his hat;" though, for the credit of the profession, we do not believe he had. By the masses, for whom we suppose it is mainly intended, the book will not be read any more than so much Choctaw; and as for the profession, Sir J. Forbes has demonstrated that they are not to be redeemed by books. In conclusion, we would recommend some glib, direct, democratic Yankee to take Sir J. Forbes' "little book and eat it up," and digest it into a fifty-page Tract for the People. We have the work from Rickey, Mallory & Co., Cincinnati.

*Mind and Matter: or, Physiological Inquiries. In a Series of Essays, intended to illustrate the Mental Relations of the Physical Organization and the Physical Faculties.* By Sir Benjamin Brodie, Bart., etc.

Dear reader, you have known talkers in whose fascinations you were held like the little bird in the charm of the snake. And so you have found talking books, that laid hold of you, and held you down, and would not let you go. This little volume is one of them. The style is most captivating in its chatty ease—running along as limpidly as a trout-brook through a summer meadow. There is no pretension in these pages; yet they are as full of "Mind and Matter" as a hive is of honey. The discussion—for the book takes the form of a pleasant conversation among three most capital friends—really adopts the metaphysics, though it ignores the technics of phrenology, and ranges through the whole wide field of anthropology. It is a book that is pleasant to read and good to study. We are indebted for a copy of the work to Rickey, Mallory & Co., Cincinnati.

---

### MISSISSIPPI VALLEY ASSOCIATION

The late meeting of this Association was, in some respects, more interesting than any one held for some years. The attendance was not so general as we have seen it. The effects of the flood on railroad traveling, no doubt, kept many away. But those who were present threw off all restraint, and went at business like brothers. Very few long speeches were made. None spoke merely to hear themselves talk; but each one seemed anxious to gain all possible information in regard to professional science. The first meeting of this Association which we attended, was in the fall of 1852—its first meeting after we became fully identified with the dental profession—and we have been at all its meetings since, and we believe that this old society has done more for our advancement in professional knowledge, than any other agency. And now, brethren, shall this pioneer association have a full meeting next year? W.

---

### CLOSE OF THE COLLEGE SESSION.

The commencement exercises of Ohio College were held in the large lecture room of the college building, which was filled to its utmost capacity. The addresses delivered on the occasion will be found in the present number of the *Register*. The names of the graduates are embodied in the minutes of the College Association. Every thing connected with the closing exercises, as well as with the meetings of the different associations, passed off pleasantly. W.

---

### CHANGE OF PROPRIETORSHIP.

Since the issue of the last No., I have, for various reasons, thought best to transfer the proprietorship and publication of the *Register* to other hands. J. T. Toland is now the publisher and proprietor of the *Dental Register*.

He has all back accounts, and it would, no doubt, be very pleasant to him to have them all liquidated immediately. The editorial charge will remain as heretofore, and, we hope, with more care and attention.

We have still to say to the profession, however, that if they wish a good journal, they must give us their aid, in contributions from the pen.

All letters pertaining to the business, or financial department, should be addressed to J. T. Toland, No. 38 West Fourth St., Cincinnati.

All communications, papers and essays, for the *Register*, should be directed to the editors of the *Dental Register*, Cincinnati, O. J. TAFT.

---

### CINCINNATI DENTAL ASSOCIATION.

We are happy to state the fact that there is, in this city, a local Dental Association, one that gives evidence of permanency. This we consider an important move, and one that "*omens*" good for the profession in our city. It was organized some seven month ago, and has been promptly attended throughout the winter, and gives promise of much good to those who attend and take part in its proceedings.

The officers are elected semi-annually, and are such as usually pertain to such organizations.

The meetings are held on the second Tuesday evening of each month, usually at the office of some member of the society. The discussions have thus far been of a thorough practical character; and at no meetings that we have ever attended, have we received so many practical items, in so short a time, as here.

Any numbers of the profession from abroad, who may be in the city on the second Tuesday evenings of any month, are most cordially invited to be present at the meetings and take part in the discussions.

In future we hope to have a synopsis of the proceedings for publication.

The next meeting will be held on the second Tuesday evening of April, at the office of Drs. Taylor & Erwin, No. 171 Race street.

*Subjects for Discussion.*—1st. Treatment of healthy pulps exposed by excavation, at a small orifice.

2d. Does living dentine possess recuperative power? T.

---

### DENTAL FURNITURE.

Mr. M. Dolph, of this city, No. 148 Walnut street, has been engaged for several years in the manufacture of Dental Furniture, such as Instrument Cases, Chairs, Spittoons, etc., which for neatness and permanency, we have not seen excelled.

He has been engaged long enough in the business to know just what the profession need, and he is always ready to furnish it, at the lowest cash price. He sells for cash, and, consequently, lower than those who sell on time. He also makes any thing in the line of show cases, neat and cheap.

